# Simultaneous Assessment of Serum Levels and Pharmacologic Effects of Cannabinoids on Endocannabinoids and *N*-Acylethanolamines by Liquid Chromatography–Tandem Mass Spectrometry

**DOI:** 10.1089/can.2021.0181

**Published:** 2023-08-09

**Authors:** Timothy A. Couttas, Carola Boost, Franziska Pahlisch, Eliska B. Sykorova, Judith E. Leweke, Dagmar Koethe, Heike Endepols, Cathrin Rohleder, F. Markus Leweke

**Affiliations:** ^1^ Brain and Mind Centre, Translational Research Collective, Faculty of Medicine and Health, The University of Sydney, Sydney, Australia.; ^2^ Department of Psychiatry and Psychotherapy, Central Institute of Mental Health, Medical Faculty Mannheim, Heidelberg University, Mannheim, Germany.; ^3^ Department of Psychiatry and Psychotherapy, University Medical Center Goettingen, Goettingen University, Goettingen, Germany.; ^4^ Department of Multimodal Imaging, Max-Planck-Institute for Neurological Research, Cologne, Germany.; ^5^ Institute of Radiochemistry and Experimental Molecular Imaging, Faculty of Medicine and University Hospital Cologne, University of Cologne, Cologne, Germany.; ^6^ Department of Nuclear Medicine, Faculty of Medicine and University Hospital Cologne, University of Cologne, Cologne, Germany.; ^7^ Forschungszentrum Juelich GmbH, Institute of Neuroscience and Medicine, Nuclear Chemistry (INM-5), Juelich, Germany.; ^8^ Endosane Pharmaceuticals GmbH, Berlin, Germany.

**Keywords:** cannabidiol, endocannabinoids, liquid chromatography–tandem mass spectrometry, *N*-acylethanolamines, rat serum, Δ^9^-tetrahydrocannabinol

## Abstract

**Introduction:**

The primary compounds of *Cannabis sativa*, delta-9-tetrahydrocannabinol (Δ^9^-THC) and cannabidiol (CBD), inflict a direct influence on the endocannabinoid system-a complex lipid signaling network with a central role in neurotransmission and control of inhibitory and excitatory synapses. These phytocannabinoids often interact with endogenously produced endocannabinoids (eCBs), as well as their structurally related *N*-acylethanolamines (NAEs), to drive neurobiological, nociceptive, and inflammatory responses. Identifying and quantifying changes in these lipid neuromodulators can be challenging owing to their low abundance in complex matrices.

**Materials and Methods:**

This article describes a robust liquid chromatography–tandem mass spectrometry (LC-MS/MS) method for the extraction and quantification of the eCBs anandamide and 2-arachidonoylglycerol, along with their congener NAEs oleoylethanolamine and palmitoylethanolamine, and phytocannabinoids CBD, Δ^9^-THC, and 11-Nor-9-carboxy-Δ^9^-tetrahydrocannabinol, a major metabolite of Δ^9^-THC. Our method was applied to explore pharmacokinetic and pharmacodynamic effects from intraperitoneal injections of Δ^9^-THC and CBD on circulating levels of eCBs and NAEs in rodent serum.

**Results:**

Detection limits ranged from low nanomolar to picomolar in concentration for eCBs (0.012–0.24 pmol/mL), NAEs (0.059 pmol/mL), and phytocannabinoids (0.24–0.73 pmol/mL). Our method displayed good linearity for calibration curves of all analytes (*R*^2^>0.99) as well as acceptable accuracy and precision, with quality controls not deviating >15% from their nominal value. Our LC-MS/MS method reliably identified changes to these endogenous lipid mediators that followed a causal relationship, which was dependent on both the type of phytocannabinoid administered and its pharmaceutical preparation.

**Conclusion:**

We present a rapid and reliable method for the simultaneous quantification of phytocannabinoids, eCBs, and NAEs in serum using LC-MS/MS. The accuracy and sensitivity of our assay infer it can routinely monitor endogenous levels of these lipid neuromodulators in serum and their response to external stimuli, including cannabimimetic agents.

## Introduction

Endocannabinoids (eCBs) and their structurally related *N*-acylethanolamines (NAEs) are endogenous lipid mediators widely distributed throughout the central nervous system (CNS) and periphery.^1^ Anandamide (AEA) and 2-arachidonoylglycerol (2-AG) are the primary eCB agonists of the endocannabinoid system (ECS), a key homeostatic system involved in neurotransmission and several regulatory processes.^2^ AEA and 2-AG act through at least two subtypes of G protein-coupled cannabinoid receptors: cannabinoid receptor 1 (CB_1_R) and cannabinoid receptor 2 (CB_2_R), which are primarily expressed in various brain regions and immune cells, respectively.^3,4^

Alterations to these eCBs have been found in several neuropsychiatric conditions, including schizophrenia,^5,6^ borderline personality and post-traumatic stress disorders,^7^ Alzheimer's,^8,9^ and Parkinson's disease.^10^ The NAEs oleoylethanolamine (OEA) and palmitoylethanolamine (PEA) exert their influence on various physiological functions, including neuroprotection, inflammation, and satiety.^11–13^

Although structurally analogous to AEA, NAE receptor signaling does not occur through CB_1/2_R but involves isoforms of the peroxisome proliferator-activated receptor family, transient receptor potential cation channel subfamily V member 1, or G protein-coupled receptor 55.^14–17^ OEA and PEA may indirectly impact CB_1/2_R-mediated signaling through their shared biosynthesis and degradation with AEA,^18^ having demonstrated the capacity to suppress AEA degradation through direct competition^19,20^ and decreased expression^21^ of fatty acid amide hydrolase (FAAH), their primary catabolic enzyme.^22^ The AEA-NAE interplay and the ubiquity of the ECS have led to increased interest of their profiles in physiology, neurology, and their response to cannabis constituents.

Exposure to delta-9-tetrahydrocannabinol (Δ^9^-THC), the principal psychoactive component of cannabis,^23^ results in overactivation of the ECS, with chronic exposure during adolescence resulting in long-lasting, potentially irreversible neurobiological alterations in various brain regions.^24^ Frequent cannabis use reduces cerebrospinal fluid AEA levels in schizophrenia and is considered a risk factor for disease development.^25^ In rodents, downregulation of AEA signaling occurs in the CNS following recurrent intraperitoneal (i.p.) injections of Δ^9^-THC.^26^ Reductions in locomotor activity and prepulse inhibition of the acoustic startle response have also been observed following acute Δ^9^-THC administration; however, the effects were dependent on the pharmacokinetic properties of the delivery solvent.^27^

Clinical trials administering the main nonpsychotomimetic compound in cannabis, cannabidiol (CBD), yield improvements in psychotic episodes without adverse side effects.^28,29^ Clinical improvement is accompanied by an increase in AEA and congener OEA and PEA ligands.^28^ This suggests that CBDs antipsychotic properties are mediated through FAAH inhibition or blockade of fatty acid-binding proteins (FABPs), which act as intracellular carriers.^30,31^ The lipophilic nature of these eCBs and NAEs also allows them to modulate and readily cross the blood–brain barrier,^32–34^ making them promising biomarker candidates and therapeutic targets.^35,36^

Quantification of eCBs and NAEs relies on mass spectrometry as their concentrations are often found at trace levels under physiological conditions, making their detection difficult.^37^ Liquid chromatography–tandem mass spectrometry (LC-MS/MS) with collision-induced dissociation (CID) is conventional for their analysis, as the additional structural information obtained from CID fragment ions can be used for selective reaction monitoring and multiple reaction monitoring (MRM) to improve sensitivity and reduce background interference.^38^ LC-MS/MS methods have been developed for eCBs,^38–42^ NAEs,^40–45^ and phytocannabinoids^46–49^ in various biological tissues. However, to the best of our knowledge, no single LC-MS/MS method has been established that monitors both endogenous and exogenous cannabinoids.

In this study we present a robust LC-MS/MS method with the capacity to reliably identify and quantify common eCBs, NAEs, and phytocannabinoids in a single assay. We successfully used our LC-MS/MS method in serum from a pharmacokinetics study on rats given i.p. injections of Δ^9^-THC or CBD to examine their associated effects on endogenous eCB and NAE lipid mediators and evaluate differential effects between pharmaceutical preparations.

## Materials and Methods

### Reagents and standards

Standards for eCBs and NAEs, including their deuterated counterparts, were purchased from Cayman Chemical (United States). Purified natural CBD and [^2^H_3_]-CBD were supplied by THC Pharm GmbH (Germany). Standards for Δ^9^-THC, 11-Nor-9-carboxy-Δ^9^-tetrahydrocannabinol (Δ^9^-THC-COOH), [^2^H_3_]-Δ^9^-THC, and [^2^H_3_]-Δ^9^-THC-COOH were provided by Lipomed AG (Switzerland). Solvents for lipid extraction and LC-MS/MS were purchased from Honeywell Specialty Chemicals (Germany).

### Calibration curves and quality controls

Twelve-point calibration curves were prepared by serial dilutions in methanol, over a concentration range of 0.012–25 pmol/mL for AEA, 0.049–100 pmol/mL for OEA/PEA, 0.244–500 pmol/mL for 2-AG, 0.122–250 pmol/mL for Δ^9^-THC/CBD, and 0.73–1500 pmol/mL for Δ^9^-THC-COOH. Calibrators were spiked with an internal standard (IS) cocktail, comprising [^2^ H_4_]-AEA (25 pmol), [^2^H_2_]-OEA and [^2^ H_4_]-PEA (100 pmol), [^2^ H_8_]-2-AG (500 pmol), [^2^ H_3_]-Δ^9^-THC (100 pmol), [^2^ H_3_]-Δ^9^-THC-COOH, and [^2^ H_3_]-CBD (250 pmol). Peak areas for each analyte were normalized against their respective deuterated IS.

Quality controls (QCs) were generated from independently prepared stock solutions at four concentrations for AEA (12.5, 1.56, 0.39, and 0.049 pmol/mL), OEA/PEA (50, 6.25, 1.56, and 0.195 pmol/mL), 2-AG (125, 31.25, 7.81, and 0.98 pmol/mL), CBD/Δ^9^-THC (250, 62.5, 15.63, and 1.95 pmol/mL), and Δ^9^-THC-COOH (750, 93.75, 23.44, and 2.93 pmol/mL). These concentrations represent the high (Q1), middle (Q2), and low (Q3) range of the calibration curves, and the lower limits of quantification (LLOQ; Q4). QCs were loaded with the same IS.

### Serum and sample extraction

Control human serum was obtained from our prior schizophrenia-related study,^25^ with rodent serum (*n*=28) obtained from investigations into behavioral changes following i.p. administration with Δ^9^-THC (5 mg/kg, *n*=14) or CBD (12 mg/kg, *n*=14) prepared in ethanol:Tween 80:saline (aqueous; with ethanol as a cosolvent and Tween-80 as surfactant, 1:1:18; *n*=8 for Δ^9^-THC, *n*=6 for CBD) or sesame oil (lipid; *n*=6 for Δ^9^-THC, *n*=8 for CBD). The Ethics Committees of the Medical Faculty Cologne, University of Cologne, Germany (00-053) and the Medical Faculty Mannheim, Heidelberg University, Germany (2009-235N-MA) approved the use of human serum samples for this research. The animal study was approved by the regional authority State Agency for Nature, Environment and Consumer Protection of the State North Rhine-Westphalia (LANIUV-NRW).

Rodent blood was withdrawn from the femoral artery 120 min postinjection and subsequently centrifuged (2054 g, 4°C) for 30 min. Serum aliquots (1 mL) were stored at −80°C until extraction. Serum aliquots were spiked with IS and extracted under chloroform/methanol (2:1, v/v).^43^ Extractions were performed at low temperatures (<4°C) to avoid artefactual AEA formation.^50^ Samples were dried under N_2_, reconstituted in methanol (80 μL), and transferred to MS vials (Brown Chromatography Supplies GmbH, Germany).

### Quantification by LC-MS/MS

LC-MS/MS was performed using an API 5000 triple quadrupole mass spectrometer (Sciex), coupled to an Agilent 1200 HPLC system (Agilent Technologies). Samples (20 μL) were injected using a CTC PAL Autosampler set at 4°C (CTC Analytics AG, Switzerland). Analytes were resolved through chromatographic separation using a 4-μm Synergi Hydro-RP C18 column (150×2 mm; Phenomenex, Torrance, CA), with column chamber set at 40°C, over a binary gradient with a flow rate of 0.5 mL/min. HPLC gradient conditions were as follows: 0 min, 25:75 A/B; 2.5 min, 20:80 A/B; 7.5 min, 10:90 A/B; 8 min, 0:100 A/B; 10 min, 25:75 A/B; 18 min, 25:75 A/B. Solvent A: 0.1% formic acid in water; Solvent B: methanol. Total run time was 18 min.

LC-MS/MS was performed in positive ion mode, [M + H]^+^, with quantifier and qualifier ion transitions selected for each analyte, at a dwell time of 50 ms. Source parameters were set as follows: positive ion spray voltage, 5000 V; ion source temperature, 500°C; collision gas, 7 psi; curtain gas, 35 psi; nebulizer gas, 25 psi; turbo gas, 45 psi. Transitions were optimized using direct infusion (10 μL/min) with each standard (100 ng/mL). MS/MS parameters are summarized in Table 1. Data were acquired and processed using Analyst^®^ (Sciex), version 1.6.2.

**Table 1.** Operational Parameters for Liquid Chromatography–Tandem Mass Spectrometry

Analyte	Precursor→product (m/z)	DP [V]	CE [V]	CXP [V]
AEA	348.3→62.0	21	25	24
	*348.3→44.0*	21	67	18
[^2^H_4_]-AEA	352.4→65.9	71	27	12
	*352.4→48.3*	71	65	10
OEA	326.4→62.0	211	27	12
	*326.4→44.0*	211	61	20
[^2^H_2_]-OEA	328.4→62.0	136	71	10
	*328.4→44.0*	136	89	18
PEA	300.3→62.0	36	23	24
	*300.3→43.9*	36	53	18
[^2^H_4_]-PEA	304.4→61.9	86	23	14
	*304.4→44.0*	86	63	18
2-AG	379.3→287.2	86	17	40
	*379.3→90.9*	86	67	24
[^2^H_8_]-2-AG	387.3→294.2	161	17	35
	*387.3→91.0*	161	68	20
CBD/Δ^9^-THC	315.3→193.1	96	31	20
	*315.3→41.0*	96	85	10
[^2^H_3_]-CBD/[^2^H_3_]-Δ^9^-THC	318.3→196.1	101	31	20
	*318.3→41.0*	101	85	10
Δ^9^-THC-COOH	345.3→327.3	176	23	14
	*345.3→299.3*	176	29	22
[^2^H_3_]-Δ^9^-THC-COOH	348.3→330.3	16	21	40
	*348.3→302.3*	16	29	42

Transitions in *italics* were used as qualifier ions.

Δ^9^-THC, delta-9-tetrahydrocannabinol; Δ^9^-THC-COOH, 11-Nor-9-carboxy-Δ^9^-tetrahydrocannabinol; 2-AG, 2-arachidonoylglycerol; AEA, anandamide; CBD, cannabidiol; CE, collision energy; CXP, collision cell exit potential; DP, declustering potential; m/z, mass to charge ratio; OEA, oleoylethanolamine; PEA, palmitoylethanolamine.

### Method validation

Our LC-MS/MS method was validated in accordance with the international requirements and regulatory guidelines for the validation of quantitative methods.^51–54^ Analyte specificity, calibration curve linearity, sensitivity, limit of detection (LOD) and LLOQ, intra- and interday accuracy, sample recovery, matrix effect, precision, and stability were assessed. Extended details on validation measures are provided (Supplementary Data).

### Statistical analysis

Serum concentrations of Δ^9^-THC, Δ^9^-THC-COOH, and CBD were compared between aqueous and lipid formulations using two-tailed, unpaired *t*-tests, corrected for multiple comparisons using the Holm–Šídák method (GraphPad Prism, version 9.1.0). Phytocannabinoid correlations with eCBs and NAEs were log-transformed (natural log) and analyzed by Pearson analysis. Grubb's test at a high stringency (*Q*=1%) was used to identify and remove a single statistical outlier for 2-AG following i.p. administered CBD with aqueous delivery formulation.

Linear regression analysis was applied to identify interaction effects between phytocannabinoid associations with eCBs and/or NAEs, using solvent delivery as the response variable, and adjusted for multiple comparisons using Bonferroni. For all experiments, statistical significance was established at *p*<0.05.

## Results

### LC-MS/MS specificity

**FIG. 1.**
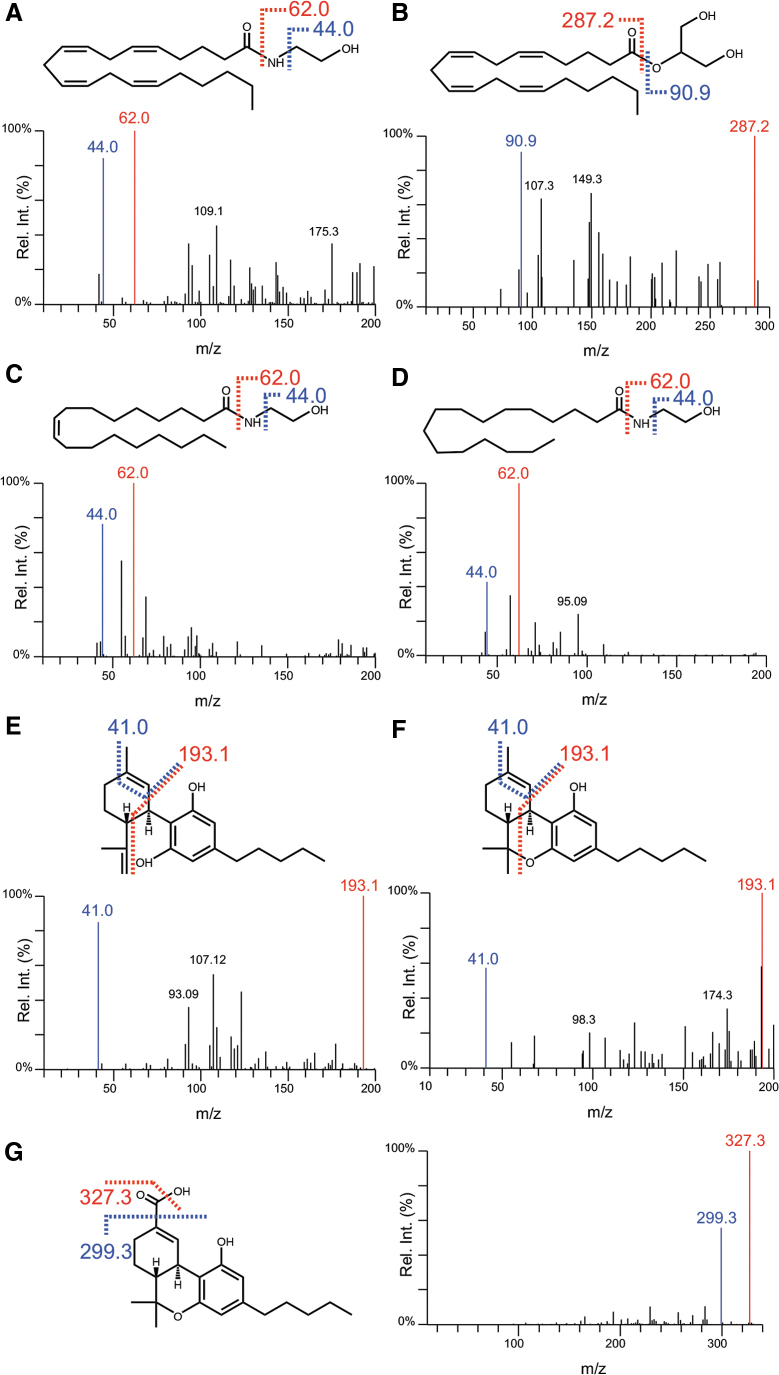
Chemical structures and MS/MS fragmentation characteristics following CID for **(A)** AEA, **(B)** 2-AG, **(C)** OEA, **(D)** PEA, **(E)** CBD, **(F)** Δ^9^-THC, and **(G)** Δ^9^-THC-COOH. Structurally diagnostic product ions used for quantitation (red) and qualifier ions (blue) have been displayed along with their expected m/z values. Δ^9^-THC, delta-9-tetrahydrocannabinol; Δ^9^-THC-COOH, 11-Nor-9-carboxy-Δ^9^-tetrahydrocannabinol; 2-AG, 2-arachidonoylglycerol; AEA, anandamide; CBD, cannabidiol; CID, collision-induced dissociation; m/z, mass to charge ratio; MS/MS, tandem mass spectrometry; OEA, oleoylethanolamine; PEA, palmitoylethanolamine.

**FIG. 2.**
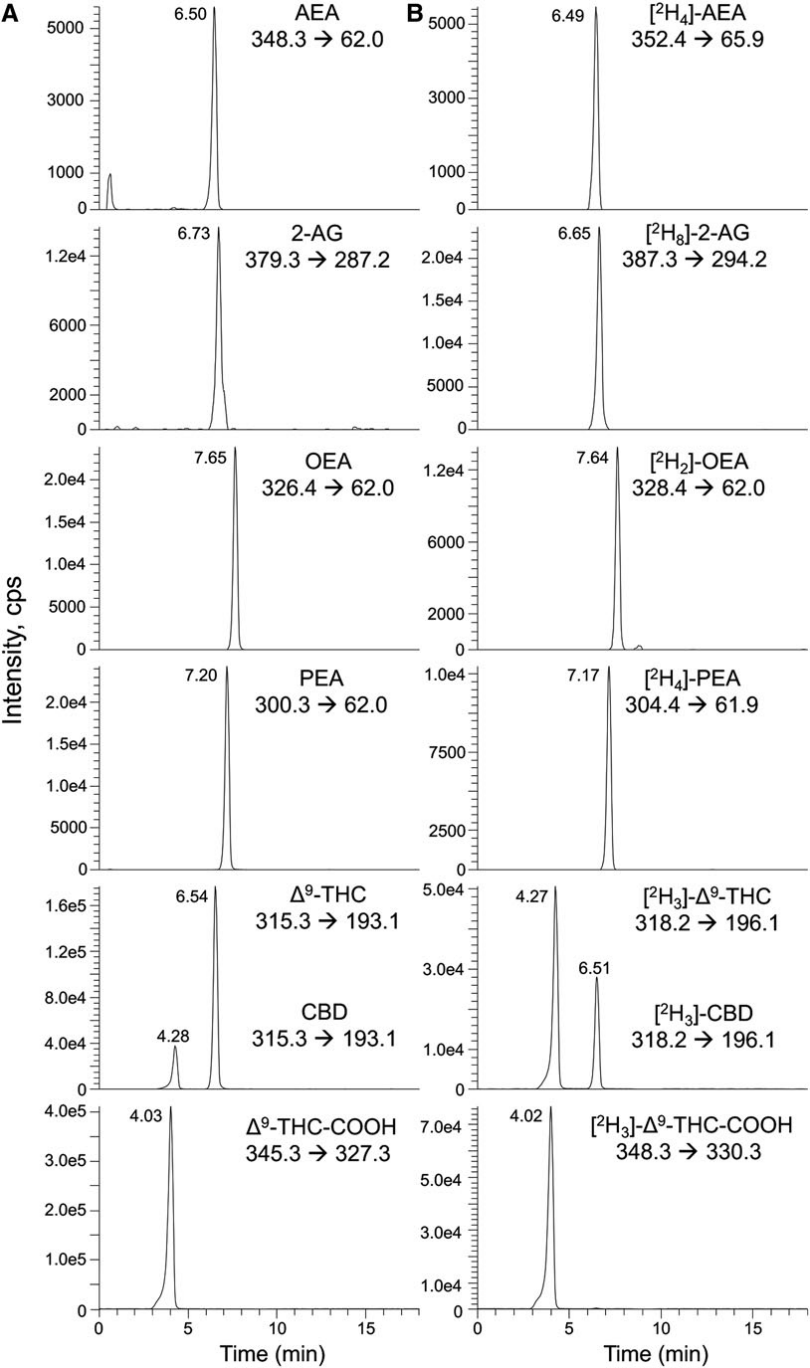
Observed chromatograms for **(A)** naturally occurring and **(B)** deuterated eCBs, NAEs, and phytocannabinoids. Peak intensity of analytes, in cps, is plotted against retention time in minutes. Resolution of isobaric Δ^9^-THC and CBD species was achieved using LC separation with a C18 column and a methanol-water gradient. cps, counts per second; eCBs, endocannabinoids; LC, liquid chromatography; NAEs, *N*-acylethanolamines.

Direct infusion of standards allowed for CID fragmentation and characterization of [M + H]^+^ ions, which were used for LC-MS/MS in MRM mode. The strongest fragment ions were selected for quantification, with the second selected as a qualifier transition. Chemical structures and fragmentation patterns of analytes are given in Figure 1. Points of fragmentation for the resultant quantifier (red) and qualifier (blue) ions are also presented. CID parameters for transitions, including IS, are given in Table 1. Quantifier/qualifier ion ratios remained within ±25% range of our reference values (data not shown). All analytes yielded unique precursor–product ion pairs, except Δ^9^-THC/CBD, which produced the same ion transitions (Table 1; Fig. 1). Their identification was resolved through chromatographic separation (Fig. 2).

LC conditions allowed for appropriate separation of all analytes, with desirable peak shape and signal intensity to perform accurate quantification (Fig. 2). AEA, 2-AG, OEA, PEA, and Δ^9^-THC-COOH were resolved at retention times (RTs) 6.50±0.01, 6.73±0.02, 7.65±0.01, 7.20±0.01, and 4.03±0.02 min, respectively, each with a relative standard deviation of <3%. Isobaric compounds CBD and Δ^9^-THC were resolved at 4.28±0.02 and 6.54±0.02 min, respectively (Fig. 2). The RTs of all IS were comparable with their naturally occurring counterparts (Fig. 2). No peaks were observed for IS in nonspiked samples (data not shown).

### Instrument sensitivity and linearity

Sensitivity of analytes was measured with respect to the LOD and LLOQ. LOD for AEA and 2-AG was 0.012 and 0.24 pmol/mL, respectively, with OEA and PEA both achieving 0.059 pmol/mL. CBD, Δ^9^-THC, and Δ^9^-THC-COOH LOD were 0.24, 0.49, and 0.73 pmol/mL, respectively. LLOQ was 0.049 for AEA, 0.195 for OEA and PEA, 0.98 for 2-AG, 1.95 for CBD and Δ^9^-THC, and 2.93 pmol/mL for Δ^9^-THC-COOH. Representative chromatograms from LLOQ have been provided (Supplementary Fig. S1).

Calibration curves (*n*=3) plotting the quotient peak area of analytes (normalized to their IS), against concentration, were constructed using a weighted linear regression analysis (*w*=1/x). Linearity was observed over the concentration range of 0.049–25 pmol/mL for AEA (*R*^2^=0.9973), 0.195–100 pmol/mL for OEA (*R*^2^=0.9990) and PEA (*R*^2^=0.9985), 0.98–250 pmol/mL for 2-AG (*R*^2^=0.9999), 1.95–250 pmol/mL for CBD (*R*^2^=0.9952) and Δ^9^-THC (*R*^2^=0.9982), and 2.93–1500 pmol/mL for Δ^9^-THC-COOH (*R*^2^=0.9901).

### Accuracy and precision

Accuracy and precision were measured across a single run (intraday) and three consecutive days (interday) through QC analyses (*n*=3). Our results show that the precision and accuracy for all analytes assayed were within our acceptance criteria, with QCs not deviating >15% (20% for the LLOQ) of the expected value (Table 2).

**Table 2.** Intraday and Interday Accuracy and Precision of Quality Controls

Analyte	QC level	Exp Conc (pmol/mL)	Intraday				Interday			
			Avg Conc (pmol/mL)	SD	Accuracy (RE, %)	Precision (RSD, %)	Avg Conc (pmol/mL)	SD	Accuracy (RE, %)	Precision (RSD, %)
AEA	QC1	12.50	14.07	0.47	12.5	3.4	14.17	0.38	13.4	2.7
	QC2	1.56	1.50	0.08	−3.8	5.3	1.60	0.07	2.6	4.4
	QC3	0.39	0.35	0.01	−9.7	1.5	0.38	0.04	−2.6	10.5
	QC4	0.05	0.05	0.00	−4.1	7.7	0.04	0.00	−9.2	9.0
2-AG	QC1	250.00	285.33	1.53	14.1	0.5	280.00	8.72	12.0	3.1
	QC2	31.25	34.43	0.06	10.2	0.2	33.30	1.10	6.6	3.3
	QC3	7.81	8.06	0.87	3.2	10.7	8.11	0.91	3.8	11.2
	QC4	0.98	1.09	0.07	11.2	6.5	1.09	0.08	11.2	7.0
OEA	QC1	50.00	49.70	3.14	−0.6	6.3	49.07	2.86	−1.9	5.8
	QC2	6.25	6.02	0.61	−3.7	10.0	5.63	0.27	−9.9	4.7
	QC3	1.56	1.39	0.03	−10.9	2.1	1.37	0.06	−12.2	4.1
	QC4	0.20	0.19	0.02	−2.6	8.9	0.20	0.02	2.6	11.5
PEA	QC1	50.00	43.51	3.30	−13.0	7.6	44.83	2.05	−10.3	4.6
	QC2	6.25	5.43	0.17	−13.1	3.2	5.34	0.25	−14.6	4.6
	QC3	1.56	1.41	0.14	−9.6	9.6	1.46	0.09	−6.4	6.3
	QC4	0.20	0.20	0.02	2.6	8.5	0.21	0.01	7.7	5.7
CBD	QC1	250.00	260.33	18.58	4.1	7.1	254.33	30.62	1.7	12.0
	QC2	31.25	28.33	1.29	−9.3	4.5	27.77	1.55	−11.1	5.6
	QC3	7.81	7.09	0.28	−9.2	3.9	7.11	0.84	−9.0	11.8
	QC4	0.98	0.94	0.08	−4.1	8.1	0.95	0.05	−3.1	5.3
Δ^9^-THC	QC1	125.00	120.00	3.46	−4.0	2.9	130.00	12.29	4.0	9.5
	QC2	31.25	27.23	2.84	−12.9	10.4	29.77	0.85	−4.7	2.9
	QC3	7.81	7.81	0.76	0.0	9.7	7.88	0.80	0.9	10.2
	QC4	1.95	1.82	0.36	−6.7	19.7	2.19	0.18	12.3	8.0
Δ^9^-THC-COOH	QC1	750.00	759.67	109.44	1.3	14.4	848.00	106.10	13.1	12.5
	QC2	93.75	105.33	3.51	12.4	3.3	105.00	3.46	12.0	3.3
	QC3	23.44	22.30	2.80	−4.9	12.6	25.37	0.92	8.2	3.6
	QC4	2.93	3.39	0.10	15.7	2.8	2.94	0.49	0.3	16.7

Accuracy was reported as percentage RE for the measured mean of spiked QCs against the nominal target value. Precision was calculated as the percentage of RSD from repeated QC measurements.

Avg Conc, average concentration from repeated QC measurements (*n*=3); Exp Conc, expected concentration; QC, quality control; RE, relative error; RSD, relative standard deviation; SD, standard deviation.

### Stability

Room temperature and 4°C were assessed by reanalyzing QCs, 24 h after initial screening. Freeze-thaw stability was measured following three cycles of QC thawing from storage conditions (24 h at −20°C). Long-term effects were examined on QCs held at −20°C for 3 months, compared with freshly prepared stocks. Analytes were predominately stable across QCs, with <15% variance from the nominal value (Table 3). Exceptions to our acceptance criteria included OEA (−15.9%), 2-AG (39.5%), and Δ^9^-THC (−18.6%) at room temperature, CBD (23.2%) and Δ^9^-THC-COOH (−33.7%) held at 4°C for 24 h. All analytes were within permissible variability following 3 months of storage at −20°C. However, inconsistencies to PEA (25.4%) and CBD (−24.2%) were observed after multiple freeze-thaw cycles.

**Table 3.** Stability Under Experimental and Storage Conditions

Analyte	Room temp			4°C			Freeze–thaw			Long-term		
	QC1	QC2	QC3	QC1	QC2	QC3	QC1	QC2	QC3	QC1	QC2	QC3
AEA	−7.5	3.5	−10.3	2.2	−10.9	−3.9	0.7	2.6	−2.6	1.8	−5.2	7.7
2-AG	1.8	−5.5	**39.5**	−1.8	−6.1	−13.5	0.0	1.7	−3.0	14.3	5.4	4.8
OEA	9.1	**−15.9**	8.0	−11.0	6.9	−2.2	−1.0	−4.2	2.8	−0.3	−2.7	5.9
PEA	5.0	−0.2	3.0	−8.7	−4.4	−5.1	−6.0	2.8	**25.4**	2.1	12.4	14.4
CBD	−7.9	−2.9	−7.5	10.0	12.8	**23.2**	**−24.2**	−11.5	13.4	14.6	1.9	−14.9
Δ9-THC	**−18.6**	3.6	−10.2	−3.3	5.3	5.8	−3.3	−0.8	−10.7	12.7	8.6	4.5
Δ9-THC-COOH	7.5	−1.8	10.3	−3.2	**−33.7**	6.3	−5.3	−3.8	11.9	13.6	8.5	9.3

Data are expressed as mean deviation (%) from initial reference. Deviations considered significant are illustrated in bold (>±15%).

### Matrix effects and sample recovery

Sample recovery and matrix effects were assessed in human serum, collected from the same healthy volunteer. Although eCBs and NAEs have been readily detected in both plasma and serum, evidence suggests their measurement is more reliable in serum owing to higher concentrations,^55,56^ which was the basis for its selection. Spiked replicates (*n*=3) were prepared for each condition (pre-extraction, postextraction, and neat methanol) using QC1, 2, and 3 concentrations as reference. Endogenous levels of eCBs and NAEs in serum were also analyzed (blank matrix, *n*=3; Supplementary Fig. S1), and their average was subtracted from spike response for a more accurate reading.

Matrix effects were deemed acceptable once normalized against their IS, with variance not exceeding 15% (Table 4). Recovery of eCBs and NAEs ranged from 87.3% to 99.8% and 76.2% to 99.7% for phytocannabinoids (Table 4). Similar yields of recovery were reported previously (82–99%) in serum^43^; however, we achieved between 2- to 10-fold higher sensitivity with the current method. 2-AG recovery was notably higher compared with prior investigations in whole blood (36.9–53.0%) and plasma (42.7%).^39,57^ Phytocannabinoid recovery corresponded to prior values for Δ^9^-THC and Δ^9^-THC-COOH (80–99%) in plasma, Δ^9^-THC-COOH in whole blood (73%) and exceeded previous yields of Δ^9^-THC (59%) and CBD (73%) extracted from whole blood, and CBD in plasma (60–70%).^47–49^

**Table 4.** Matrix Effect and Analyte Recovery

Analyte	Matrix effect			Normalized matrix effect			Sample recovery		
	QC1	QC2	QC3	QC1	QC2	QC3	QC1	QC2	QC3
AEA	11.4	14.8	13.6	7.1	7.9	2.9	89.8	99.8	99.1
OEA	24.0	16.2	20.9	9.2	7.1	3.7	91.1	88.9	87.3
PEA	8.7	17.6	28.2	5.4	7.6	14.9	96.4	96.6	95.3
2-AG	21.1	20.5	34.6	9.7	5.8	14.4	91.6	98.0	94.2
CBD	13.5	10.1	27.2	5.1	3.4	10.2	95.2	99.7	97.9
Δ^9^-THC	12.2	18.9	11.7	5.8	9.2	8.3	84.1	99.3	93.5
Δ^9^-THC-COOH	11.4	14.8	13.6	5.3	7.9	6.7	76.2	79.7	85.6

Values are expressed as mean % (*n*=3).

### Monitoring eCB/NAE expression against administered phytocannabinoid concentration

**FIG. 3.**
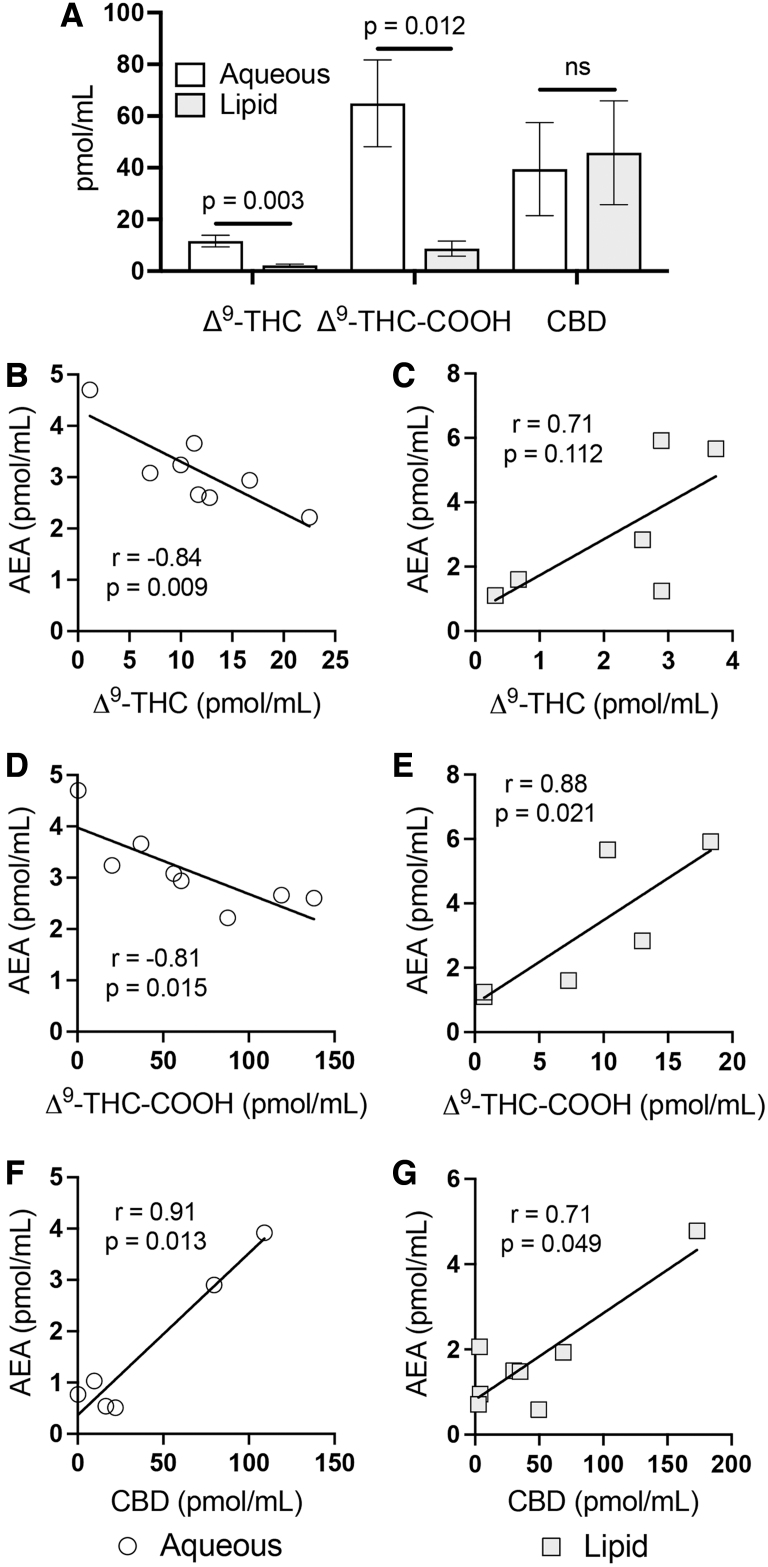
Effects on AEA in serum following Δ^9^-THC and CBD i.p. administration. **(A)** Concentrations of Δ^9^-THC, Δ^9^-THC-COOH, and CBD in serum 120 min after i.p. injection (5 mg/kg Δ^9^-THC; 12 mg/kg CBD). Results show mean and standard error for different delivery solvents (aqueous and lipid). A statistically significant difference in Δ^9^-THC (*p*=0.003) and Δ^9^-THC-COOH (*p*=0.012) between aqueous and lipid solvents was identified by unpaired *t*-tests. **(B–G)** display correlations for AEA with **(B, C)** Δ^9^-THC, **(D, E)** Δ^9^-THC-COOH, and **(F, G)** CBD when either **(B, D, F)** aqueous or lipid-based **(C, E, G)** solvent formulations were used. A line of best fit (linear regression) is shown. Correlations were determined by Pearson analysis at a confidence interval of 95%. The coefficient of correlation (*r*) and *p*-values are given. i.p., intraperitoneal; ns, not significant.

Our LC-MS/MS method was used to measure eCBs and NAEs against phytocannabinoid concentrations in rat serum following i.p. injections with Δ^9^-THC and CBD, prepared in both aqueous and lipid-based formulations for delivery comparison. Aqueous formulation yielded significantly greater serum levels of Δ^9^-THC (*p*=0.003), and its metabolite Δ^9^-THC-COOH (*p*=0.012), than the lipid formulation 120 min postinjection, whereas no discernible differences to CBD levels were observed between delivery solvents (Fig. 3A). Solvent properties appear to have also influenced Δ^9^-THC associations with eCBs and NAEs, as each eCB/NAE correlation was contrary between the two formulations (Table 5).

**Table 5.** Phytocannabinoid Correlations with Endocannabinoids and *N*-Acylethanolamines

	Analyte	AEA	OEA	PEA	2-AG
Δ^9^-THC (aqueous)	*r*	**−0.8432**	−0.0073	−0.4161	0.4411
	*p*	**0.0085**	0.9864	0.3051	0.2739
Δ^9^-THC (lipid)	*r*	0.7121	0.6188	0.6164	−0.3188
	*p*	0.1124	0.1903	0.1925	0.5381
Δ^9^-THC-COOH (aqueous)	*r*	**−0.8088**	−0.3740	−0.0041	0.4302
	*p*	**0.0151**	0.3614	0.9923	0.2874
Δ^9^-THC-COOH (lipid)	*r*	**0.8803**	0.6244	**0.8657**	−0.7356
	*p*	**0.0206**	0.1852	**0.0259**	0.0956
CBD (aqueous)	*r*	**0.9043**	0.7483	0.7610	0.7858
	*p*	**0.0133**	0.0871	0.0789	0.0639
CBD (lipid)	*r*	**0.7079**	0.7036	0.5172	−0.1244
	*p*	**0.0495**	0.0515	0.1893	0.7692

Pearson correlation analysis was used to determine associations between exogenous (Δ^9^-THC, CBD) and endogenous (AEA, OEA, PEA, 2-AG) cannabinoids (log-transformed). Coefficients of correlation (*r*) and *p*-values are given, with significance illustrated in bold.

Linear regression verified significant opposing associations between the delivery solvents for Δ^9^-THC (*p*=0.006) and Δ^9^-THC-COOH (*p*<0.0001) with AEA (Fig. 3B–E), which were the result of significantly negative relationships with aqueous Δ^9^-THC administration (Δ^9^-THC-AEA: *r*=−0.84, *p*=0.009; Δ^9^-THC-COOH-AEA: *r*=−0.81, *p*=0.015) and positive associations with lipid-based delivery (Δ^9^-THC-AEA: *r*=0.71, *p*=0.112; Δ^9^-THC-COOH-AEA: *r*=0.88, *p*=0.021). CBD interactions with AEA and its congeners were consistent between delivery formulations (Fig. 3E, F; Table 5).

Pearson analysis showed a significantly positive association for CBD-AEA in both aqueous (*r*=0.91, *p*=0.013) and lipid preparations (*r*=0.71, *p*=0.049). Positive trends were also observed with OEA (aqueous: *r*=0.74, *p*=0.087; lipid: *r*=0.70, *p*=0.052) and PEA (aqueous: *r*=0.76, *p*=0.080; lipid: *r*=0.61, *p*=0.113). CBD exhibited no significant association with 2-AG when either aqueous (*r*=0.23, *p*=0.706) or lipid-based (*r*=−0.15, *p*=0.721) formulation was used.

## Discussion

This study presents an LC-MS/MS method capable of accurately quantifying endogenous and exogenous cannabinoids in a single assay with comparable^39,47,49^ or greater sensitivity^40–42,44–46,48^ to prior approaches. Our method was successfully applied to monitor endogenous levels of eCBs and NAEs in human serum and showed its capacity to measure their associations in response to Δ^9^-THC or CBD injections in rats. Our findings support prior clinical evidence that CBD positively regulates AEA levels, along with its congeners in serum.^28^

Future examination is warranted to assess whether the mechanism responsible for the observed CBD-AEA/NAE trends in our data are a consequence of CBDs capacity to inhibit their shared FAAH hydrolysis and FABPs-mediated intracellular transport. Although positive correlations were also observed with OEA and PEA, the degree of association was not as significant, suggesting the underlying mechanism may be more complex than blockage of their shared degradation pathway.

Δ^9^-THC correlations with eCBs and NAEs were more confounding, particularly the opposing directionality with different delivery formulations. Previously, we highlighted only aqueous i.p. injections of Δ^9^-THC elicit behavioral abnormalities in rats owing to a faster kinetic than the lipid formulation, most likely owing to a different amount of CB_1_R activation per time unit.^27^ The relationship between the speed of drug delivery and physiological and neuropsychological effects has also been observed in studies analyzing the effects of drugs such as cocaine or methylphenidate. Thereby, the time to peak effect had been suggested to be critical for the reward effects, possibly because of rapid changes in dopamine release.^58–60^

We speculate a faster rate of Δ^9^-THC accrual with the aqueous solvent may explain its five-fold higher concentration in serum compared with the lipid formulation, and the significant inverse association observed with AEA that aligns with prior literature.^25,26^

Δ^9^-THC-COOH response corroborates our Δ^9^-THC findings, as this metabolite is used as an indicator of cannabis consumption owing to Δ^9^-THC rate of oxidation and stability of Δ^9^-THC-COOH,^48^ and its reported association with low AEA at high concentrations.^61^ Impact on AEA may be a consequence of Δ^9^-THC-CB_1_R-activated AEA membrane transport^62^ for intracellular degradation by FAAH.^63^ Alternatively, AEA synthesis has been described as an “on-demand” process linked to its receptor coupling.^2^ As Δ^9^-THC and AEA are both CB_1_R-selective agonists,^22^ negative feedback through competitive CB_1_R binding may impact AEA production. This latter mechanism may explain why similar effects on OEA and PEA were not observed.

To the best of our knowledge, this is the first reported application of LC-MS/MS that combines exogenous and endogenous cannabinoid assessment in a single assay. Given our assay can routinely measure low concentrations of these lipid mediators in serum and the versatility of the LC conditions used, our assay has the potential to be extended to other biological matrices following adaptations to the extraction procedure, if necessary.

Although our method boasts several developments, care must still be taken to avoid interference by ion suppression, especially when switching between matrices (e.g., whole blood, plasma, and brain tissue) that display different levels of recovery.^39–49^ 2-AG quantification is additionally complicated by its spontaneous isomerization to 1-AG, particularly evident in polar solvents that have a higher degree of acyl migration. Because of inconstant levels of expression, effects of 1-AG isomerization were not examined in this study, although their separation was feasible (Supplementary Fig. S1).

Cannabinoid instability is also a risk, as degradation and adherence to plastics are common and result in poor detection and unreliable quantitation.^64,65^ We recommend samples to be stored at a minimum of −20°C, aliquoted to prevent freeze–thawing with multiple analyses, and assessed within 24 h to reduce the likelihood of inconsistent results. It should be noted that stability measurements were used on QCs, owing to limited sample availability, which should be acknowledged as a limitation. Nevertheless, we have previously shown the stability of these endogenous analytes in serum when extracted under the same procedure.^43^ Consideration should also be given to the “delivery” of cannabimimetic agents to ensure that the appropriate response is achieved.

## Supplementary Material

Supplemental data

Supplemental data

## Abbreviations Used

Δ^9^-THC: delta-9-tetrahydrocannabinol
Δ^9^-THC-COOH: 11-Nor-9-carboxy-Δ^9^-tetrahydrocannabinol
2-AG: 2-arachidonoylglycerol
AEA: anandamide
CB_1_R: cannabinoid receptor 1
CB_2_R: cannabinoid receptor 2
CBD: cannabidiol
CE: collision energy
CID: collision-induced dissociation
CNS: central nervous system
cps: counts per second
CXP: collision cell exit potential
DP: declustering potential
eCBs: endocannabinoids
ECS: endocannabinoid system
FAAH: fatty acid amide hydrolase
FABPs: fatty acid-binding proteins
i.p.: intraperitoneal
IS: internal standard
LC-MS/MS: liquid chromatography–tandem mass spectrometry
LLOQ: lower limits of quantification
LOD: limit of detection
m/z: mass to charge ratio
MRM: multiple reaction monitoring
NAEs: *N*-acylethanolamines
OEA: oleoylethanolamine
PEA: palmitoylethanolamine
QCs: qquality controls
RE: relative error
RSD: relative standard deviation
RTs: retention times
SD: standard deviation
