# Supplementary material for: Simultaneous Assessment of Serum Levels and Pharmacologic Effects of Cannabinoids on Endocannabinoids and N-Acylethanolamines by Liquid Chromatography–Tandem Mass Spectrometry
Source: Cannabis Cannabinoid Res. 2023 Aug 9;8(4):657–69. doi: 10.1089/can.2021.0181 (PMC10442685; doi:10.1089/can.2021.0181)
Supplement: Supplemental data [file Suppl_Data.docx]

**Supplementary Information**

**S1: Extended details on LC-MS/MS method validation procedures**

***Instrument sensitivity and linearity***

The limit of detection (LOD) was defined as the lowest concentration of analyte whose signal can be reliably differentiated from background noise (S/N value ≥ 3). The lower limit of quantification (LLOQ) was defined at the lowest concentration that can be quantitatively determined with suitable precision and accuracy, S/N ≥ 10). Linearity of calibration curves were deemed suitable if the concentrations for each calibrator did not deviate by more than 15% from the stated value (20% for the LLOQ) and maintained a coefficient of determination (R^2^) > 0.99.

***Accuracy and Precision***

Accuracy was reported as percentage relative error for the measured mean from spiked QCs against their nominal target value. Precision was calculated as the percentage of relative standard from repeated QC measurements.

***Stability***

Stability values were expressed as a percentage coefficient of the deviation between condition and reference value against the mean. Reported values were deemed acceptable if they did not deviate more than 15% from the expected value.

***Matrix Effects***

Matrix effect was calculated as the % difference between peak area of serum spiked with analytes post-extraction, against analytes spiked directly in methanol. Triplicate analyses were performed with analytes using concentrations at QCs 1, 2, and 3 as reference. In addition, the normalized matrix effects were also examined, defined as the observed variability between the two conditions (post-extraction and neat solvent) when analytes are normalized to their respective deuterated standard. This normalized matrix effect was deemed significant if variability was above 15%.

***Sample Recovery***

Sample recovery was assessed serum specimens from the same healthy male subject. Serum was spiked at three different concentrations (QC1, 2, and 3) with naturally occurring standards and corresponding deuterated internal standards. Recovery of each analyte following extraction was assessed serum spiked with same analyte concentration pos-extraction.
