# Supplementary material for: Simultaneous Assessment of Serum Levels and Pharmacologic Effects of Cannabinoids on Endocannabinoids and N-Acylethanolamines by Liquid Chromatography–Tandem Mass Spectrometry
Source: Cannabis Cannabinoid Res. 2023 Aug 9;8(4):657–69. doi: 10.1089/can.2021.0181 (PMC10442685; doi:10.1089/can.2021.0181)

**Supplementary figure 1.** Representative chromatograms for (A) AEA, (B) 2-AG, (C) OEA, (D) PEA, (E) CBD and Δ^9^‐THC, and Δ^9^-THC-COOH (F) at the LLOQ (black) and analysis of blank extracted control serum (red).


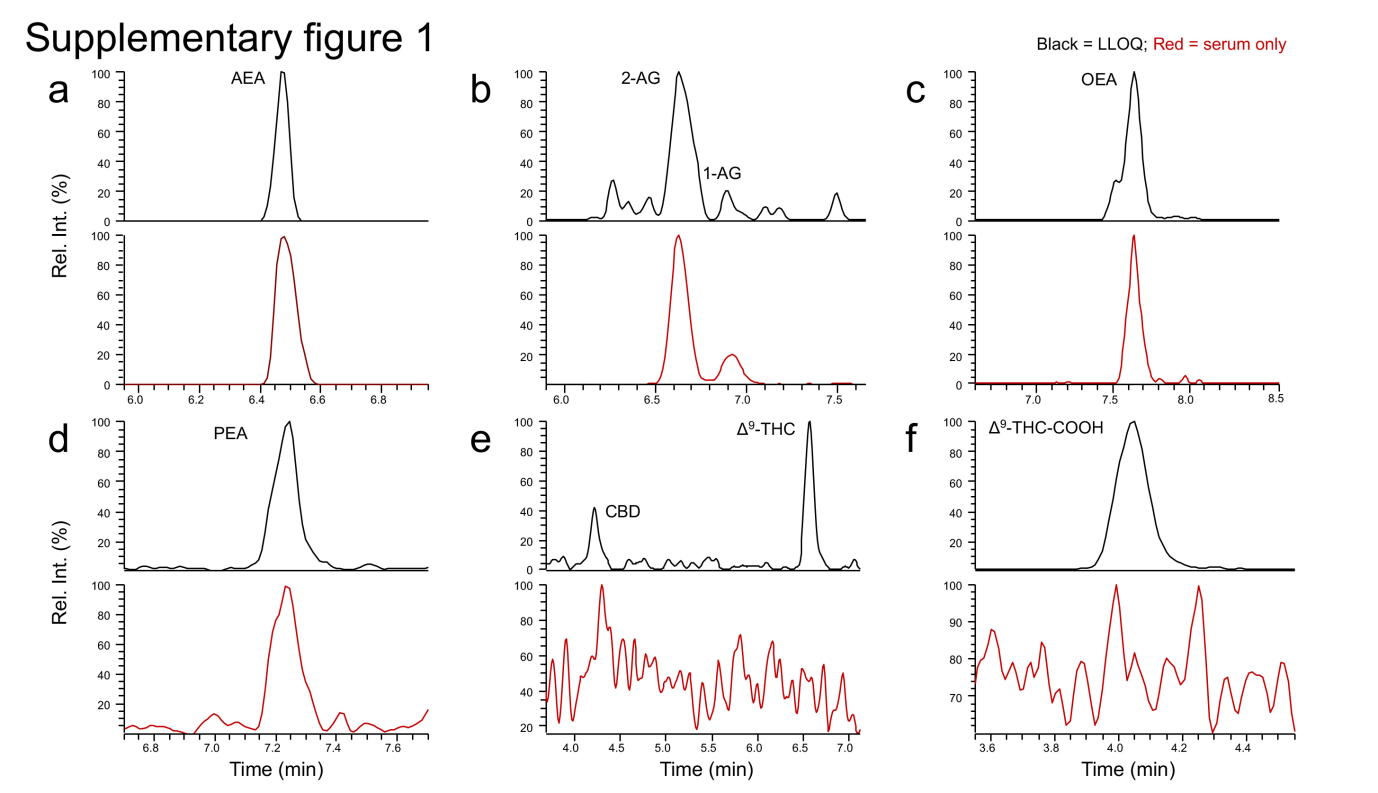

Supplement: Supplemental data [file Suppl_FigureS1.docx]
